# Systematic metabolic characterization of mental disorders reveals age‐related metabolic disturbances as potential risk factors for depression in older adults

**DOI:** 10.1002/mco2.165

**Published:** 2022-09-30

**Authors:** Yu Liu, Wanyu Zhao, Ying Lu, Yunli Zhao, Yan Zhang, Miao Dai, Shan Hai, Ning Ge, Shuting Zhang, Mingjin Huang, Xiaohui Liu, Shuangqing Li, Jirong Yue, Peng Lei, Biao Dong, Lunzhi Dai, Birong Dong

**Affiliations:** ^1^ National Clinical Research Center for Geriatrics and Department of General Practice State Key Laboratory of Biotherapy West China Hospital Sichuan University Chengdu China; ^2^ Department of Health Research Methods, Evidence, and Impact McMaster University Hamilton Ontario Canada; ^3^ Department of Neurology, West China Hospital Sichuan University Chengdu China; ^4^ The Third Hospital of Mianyang Sichuan Mental Health Center Mianyang China; ^5^ School of Life Sciences Tsinghua University Beijing China

**Keywords:** aging, mental illness, metabolomics, lipidomics, molecular classification

## Abstract

Mental disorders are associated with dysregulated metabolism, but comprehensive investigations of their metabolic similarities and differences and their clinical relevance are few. Here, based on the plasma metabolome and lipidome of subcohort1, comprising 100 healthy participants, 55 cases with anxiety, 52 persons with depression, and 41 individuals with comorbidity, which are from WCHAT, a perspective cohort study of community‐dwelling older adults aged over 50, multiple metabolites as potential risk factors of mental disorders were identified. Furthermore, participants with mental illnesses were classified into three subtypes (S1, S2, and S3) by unsupervised classification with lipidomic data. Among them, S1 showed higher triacylglycerol and lower sphingomyelin, while S2 displayed opposite features. The metabolic profile of S3 was like that of the normal group. Compared with S3, individuals in S1 and S2 had worse quality of life, and suffered more from sleep and cognitive disorders. Notably, an assessment of 6,467 individuals from the WCHAT showed an age‐related increase in the incidence of depression. Seventeen depression‐related metabolites were significantly correlated with age, which were validated in an independent subcohort2. Collectively, this work highlights the clinical relevance of metabolic perturbation in mental disorders, and age‐related metabolic disturbances may be a bridge‐linking aging and depressive.

## Graphical abstract

Based on plasma metabolomics and lipidomics, we identified numerous potential risk factors for older adults with anxiety, depression, or comorbid anxiety‐depressive symptoms. Molecular classification revealed the clinical relevance of metabolic perturbations associated with these mental disorders. In addition, the incidence of depression increases with age. We revealed that the changes of depression‐related metabolites were significantly correlated with age, indicating the interplay among age, metabolic disturbances, and depression.

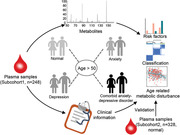

## 1 INTRODUCTION

Mental disorders, also called mental illnesses or psychiatric disorders, are common, although often ignored diseases, and approximately 29% of the population suffers from a mental illness at least once in their lifetime.^1^ Diverse types of mental disorders with distinct behaviors and personalities have been identified.^1^, ^2^ Among them, depression and anxiety are two of the most prevalent mental disorders in older adults above age 55 and belong to the top 20 causes of disability worldwide.^3^ Over 50% of older adults with depression also have concurrent symptoms of anxiety.^4^, ^5^ Comorbid anxiety‐depressive disorder is usually linked to more severe symptoms, more frequent functional disability,^6^ higher suicide risk,^7^ and even a higher mortality rate.^8^

The current diagnosis of anxiety, depression, and comorbid depressive disorder is usually based on questionnaires, in which a set of symptoms of the patients are assessed by doctors or psychiatrists.^9^, ^10^ However, due to the unclear boundaries between mental disorders, some people may be overdiagnosed, underdiagnosed, or even misdiagnosed.^11^, ^12^ Social support, quality of life, and comorbid diseases were usually considered in the interviews, but not enough. The state‐of‐the‐art functional near‐infrared spectroscopy (fNIRS) and machine learning have recently been used in the assessment of various psychiatric disorders.^13^ Besides, molecular classification using omics data has emerged as a robust tool for disease diagnosis and assessment.^14^, ^15^, ^16^ The changes in some metabolites serve as biomarkers or driving forces of depressive and anxiety disorders.^17^, ^18^ For example, high‐fat diet, type 2 diabetes mellitus, obesity, and insulin resistance are associated with the incidence and progression of mental disorders, and some of these metabolic issues further affect the antidepressant treatment.^19^ In addition, the alterations of amino acid, energy, and lipid metabolites are also closely linked to the depression and anxiety disorders.^20^, ^21^, ^22^, ^23^ Therefore, metabolic classification of mental disorders may make the diagnosis of mental disorders more accurate.

Peripheral pro‐inflammatory cytokines, such as interleukin 6 (IL‐6), interleukin 17 (IL‐17), tumor necrosis factor alpha (TNF‐α) and C‐reactive protein (CRP), significantly increase in depressive patients,^24^, ^25^ but dramatically decrease after the treatment of antidepressant^26^. Many studies have shown that systemic cytokine levels are remodeled with age and tend toward a proinflammatory phenotype called inflammaging.^27^ Inflammaging is one of the main causes of many diseases, including organ aging, cancer, neurodegenerative diseases, and psychiatric disorders.^28^ It is further exacerbated by redox imbalance, senescence‐associated secretory phenotype (SASP) and a decline in effective autophagy with age.^29^ Abnormal cellular metabolism is a hallmark of aging.^30^ Lipid accumulation, impaired glucose metabolism and insulin sensitivity, decreased systemic NAD^+^ and sirtuin activity, and cellular antioxidant deficiencies are known metabolic disorders associated with aging.^31^ Previous studies have shown that the metabolic disturbances are closely associated with the incidence and progression of psychiatric disorders.^20^, ^21^, ^22^, ^23^, ^32^ However, their metabolic similarities and differences have rarely been investigated. In addition, the associations between aging, metabolic disturbances, and mental disorders remain largely unknown.

**FIGURE 1:**
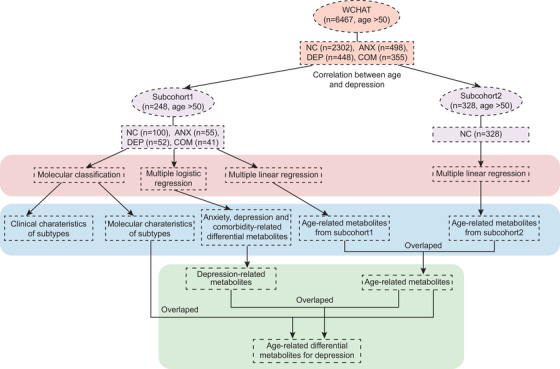
Experimental design. A total of 576 plasma samples were selected from the West China Health and Aging Trend study (WCHAT) cohort (> 50 years old) and divided into two subcohorts for metabolomics and lipidomics profiling. Subcohort1 included 248 individuals, 148 of whom had anxiety, depressive, or comorbid anxiety–depressive symptoms, diagnosed by two doctors based on the GDS‐15 and GAD‐7 questionnaires, and 100 who were mentally healthy. Subcohort2 had 328 individuals without mental disorders. NC, normal control; ANX, anxiety; DEP, depression; COM, comorbid anxiety‐depressive; GDS‐15: Geriatric Depression Scale‐15; GAD‐7: General Anxiety Disorder‐7

To answer these questions, we performed metabolomics and lipidomics analyses of 248 older adults in the West China Health and Aging Trend study (WCHAT) cohort, a prospective cohort comprising of 7,439 participants aged over 50 years from West China,^33^ to disclose the potential metabolic risk factors and the metabolic subtypes of anxiety, depression, and comorbid anxiety‐depressive disorder. A close relationship between metabolic profiles and clinical phenotypes was observed. In addition, we confirmed the association between age and depression using 6,467 individuals with mental health assessment data from the WCHAT cohort. Besides, we screened the metabolic changes associated with age and depressive symptoms using subcohort1 (*n* = 248) from WCHAT cohort. The association of these metabolites with age was validated in an independent subcohort2 with 328 mental health participants from WCHAT cohort (Figure 1). This study clarified the metabolic similarities and differences of anxiety, depression, and comorbid depressive disorder, build tighter connections between the severity of mental disorders and metabolic profiles, and revealed the age‐related metabolic disturbances for depression.

## 2 RESULTS

### 2.1 Characteristics of the participants in this study

**FIGURE 2:**
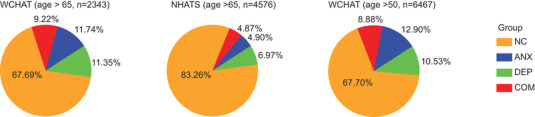
Prevalence of anxiety, depression, and comorbid anxiety–depressive disorder in WCHAT. The incidence of anxiety, depression, and comorbidity in the West China Health and Aging Trend study (WCHAT) and National Health Aging Trends Study (NHATS) cohorts

Prevalence analysis of mental health assessments of 2,343 WCHAT participants over 65 showed that 11.74%, 11.35%, and 9.22% of them suffered from anxiety, depression, and comorbid anxiety‐depressive disorder, respectively. The incidence was much higher than that in the National Health Aging Trends Study (NHATS) of the United States (the participants aged over 65) (Figure 2).^34^ In addition, people over age 65 were more likely to suffer from depressive symptoms than those over age 50 (11.35% vs. 10.53%, Figure 2). To profile the plasma metabolome associated with anxiety, depression, and comorbid anxiety‐depressive disorder, a total of 248 (subcohort1) WCHAT participants, including 41 individuals with comorbid anxiety and depression, 52 persons with depression, 55 cases with anxiety, and 100 control participants, were randomly selected. The persons who have used antidepressants were not included in the subcohort1. The mental health status of 248 participants was diagnosed by clinicians using the Geriatric Depression Scale (GDS‐15) and General Anxiety Disorder (GAD‐7) questionnaires, and the characteristics of 248 participants are summarized in Table 1. No significant differences in age, sex, education, marital status, smoking, or drinking were observed among the four groups of participants, but the average body mass index (BMI) of comorbid patients was significantly lower (ANOVA test, *p* < 0.05), consistent with the Hamilton Rating Scale for Depression (HRSD).^35^

**TABLE 1:** Clinical characteristics of participants in this study

	**Subcohort1**					**Subcohort2**
	**Control**	**Anxiety**	**Depression**	**Comorbid**		**Normal**
	**(*N* = 100)**	**(*N* = 55)**	**(*N* = 52)**	**(*N* = 41)**	***p*‐Value**	**(*N* = 328)**
GDS score^a^, mean (SD)	0.5 (0.5)	2.5 (1.2)	6.3 (1.6)	7.7 (2.2)	< 0.001	2.0 (1.2)
GAD score^a^, mean (SD)	0.1 (0.3)	7.5 (2.6)	1.9 (1.6)	8.9 (4.2)	< 0.001	1.1 (1.3)
Age^a^, mean (SD)	68.8 (10.0)	69.6 (8.3)	68.7 (9.9)	66.1 (9.3)	0.33	68.5 (8.7)
Sex^b^, No. (%)					0.27	
Female	62 (62.0%)	38 (69.1%)	39 (75.0%)	31 (75.6%)		201 (61.3%)
Male	38 (38.0%)	17 (30.9%)	13 (25.0%)	10 (24.4%)		127 (38.7%)
Education^b^, No. (%)					0.39	
Illiterate	27 (27.0%)	21 (38.2%)	21 (40.4%)	13 (31.7%)		104 (31.7%)
Primary school	40 (40.0%)	19 (34.5%)	21 (40.4%)	13 (31.7%)		129 (39.3%)
Secondary school and above	33 (33.0%)	15 (27.3%)	10 (19.2%)	15 (36.6%)		95 (29.0%)
Marital status^b^, No. (%)					0.97	
Married	71 (71.0%)	41 (74.5%)	37 (71.2%)	30 (73.2%)		69 (21.0%)
Unmarried/widowed/divorced	29 (29.0%)	14 (25.5%)	15 (28.8%)	11 (26.8%)		259 (79.0%)
Smoking^b^, No. (%)					0.44	
No	79 (79.0%)	46 (83.6%)	44 (88.0%)	36 (87.8%)		251 (76.8%)
Yes	21 (21.0%)	9 (16.4%)	6 (12.0%)	5 (12.2%)		76 (23.2%)
Drinking wine^b^, No. (%)					0.33	
No	85 (85.0%)	44 (80.0%)	48 (92.3%)	34 (82.9%)		251 (76.5%)
Yes	15 (15.0%)	11 (20.0%)	4 (7.69%)	7 (17.1%)		77 (23.5%)
BMI^a^,						
mean (SD)	23.1 (3.1)	24.1 (3.6)	23.1 (3.5)	21.8 (3.0)	0.009	1.1 (1.3)

^a^ Continuous variables, ANOVA test.

^b^ Categorical variables, Chi‐squared test. GDS: Geriatric Depression Scale; GAD: General Anxiety Disorder; BMI: body mass index, calculated as weight in kilograms divided by height in meters squared.

### 2.2 Metabolic risk factors for anxiety, depression, and comorbid anxiety‐depressive disorder

**FIGURE 3:**
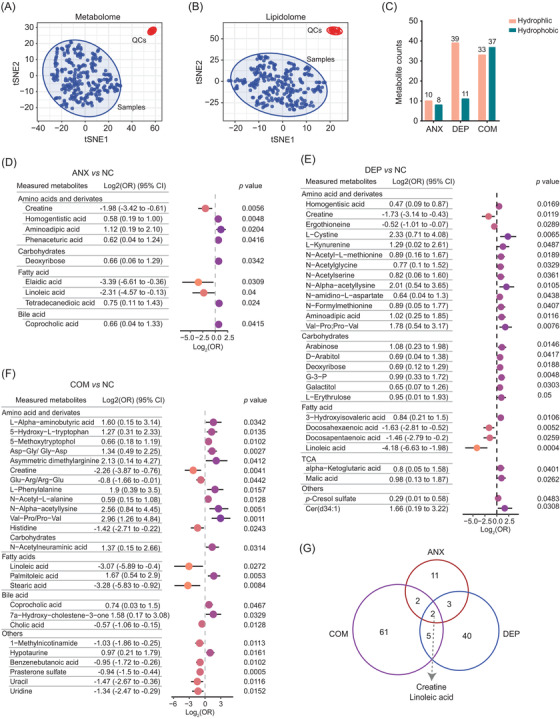
The metabolites with significant differences in anxiety, depression, and comorbid anxiety–depressive disorder. (A,B) T‐SNE analysis of the metabolome (A) and lipidome (B) of samples and QCs. (C) Statistics of the significantly differential metabolites of anxiety, depression, and comorbid anxiety–depressive disorder. (D–F) Forest plots illustrate pooled log2 transformed OR and 95% CI for the main differential metabolites (multiple logistic regression, *p* < 0.05) in anxiety (D), depression (E), and comorbidity (F). Logistic regression was adjusted by age, sex, and body mass index (BMI). (G) The overlap of differential metabolites among the anxiety, depression, and comorbidity groups. QCs, quality controls; NC, normal control; ANX, anxiety; DEP, depression; COM, comorbid anxiety–depressive; OR, odds ratio; CI, confidence interval

To reveal the metabolic risk factors for anxiety, depression, and comorbid anxiety‐depressive disorder, we next carried out untargeted metabolomics and lipidomics profiling of the 248 plasma samples. In total, 576 metabolites were identified by mass spectrometry (MS). T‐SNE analysis of metabolomics data, lipidomics data, and quality controls (QCs) indicated high data quality (Figure 3A,B). To uncover the potential metabolic risk factors for depressive symptoms, multiple logistic regression analyses were performed on metabolomic and lipidomic data.^36^ Correlations were measured with odd ratios (ORs). In total, 18, 50, and 70 metabolites were identified as risk factors for anxiety, depression, and comorbid anxiety‐depressive disorder, respectively (*p* < 0.05, Figure 3C–F). The more severe the mental disorder was, the more metabolic risk factors were identified.

Many metabolites, including docosapentaenoic acid (DPA, log_2_(OR) = −1.46, 95% CI: −2.79 to −0.2, *p* = 0.0259), docosahexaenoic acid (DHA, log_2_(OR) = −1.63, 95% CI: −2.81 to −0.52, *p* = 0.0052), and ergothioneine (log_2_(OR) = −0.52, 95% CI: −1.01 to −0.07, *p* = 0.0289), are known to benefit depressive symptoms and decrease in the depression group (Figure 3E).^37^, ^38^ In addition, amino acids and their derivatives are the main differential metabolites for depressive symptoms, such as homogentisic acid (log2(OR) = 0.47 95% CI: 0.09–0.87, *p* = 0.0169), L‐cystine (log_2_(OR) = 2.33, 95% CI: 0.71–4.08, *p* = 0.0065), and L‐kynurenine (log2(OR) = 1.29, 95% CI: 0.02–2.61, *p* = 0.0487) (Figure 3E). In comorbid participants, hydrophilic metabolites such as prasterone sulfate (log2(OR) = −0.94, 95% CI: −1.5 to −0.44, *p* = 0.0005), 1‐methylnicotinamide (log2(OR) = −1.03, 95% CI: −1.86 to −0.25, *p* = 0.0113), benzenebutanoic acid (log2(OR) = −0.95, 95% CI: −1.72 to −0.26, *p* = 0.0102), and uracil (log2(OR) = −1.47, 95% CI: −2.67 to −0.36, *p* = 0.0116) were significantly reduced (Figure 3F). Overlapping analysis revealed that two metabolites were simultaneously reduced in anxiety, depression, and comorbidity, including creatine (depression: log_2_(OR) = −1.73, 95% CI: −3.14 to −0.43, *p* = 0.0119; anxiety: log_2_(OR) = −1.98, 95% CI: −3.42 to −0.605, *p* = 0.0056; comorbidity: log_2_(OR) = −2.26, 95% CI: −3.87 to −0.76, *p* = 0.0041) and linoleic acid (depression: log_2_(OR) = −4.21, 95% CI: −6.63 to −1.98, *p* = 0.0004; anxiety: log_2_(OR) = −2.31, 95% CI: −4.57 to −0.13, *p* = 0.04; comorbidity: log_2_(OR) = −3.07, 95% CI: −5.89 to −0.4, *p* = 0.0272) (Figure 3G). Collectively, we present many potential metabolic risk factors for anxiety, depression, and comorbid anxiety‐depressive disorder.

### 2.3 Molecular stratification of individuals with mental disorders

**FIGURE 4:**
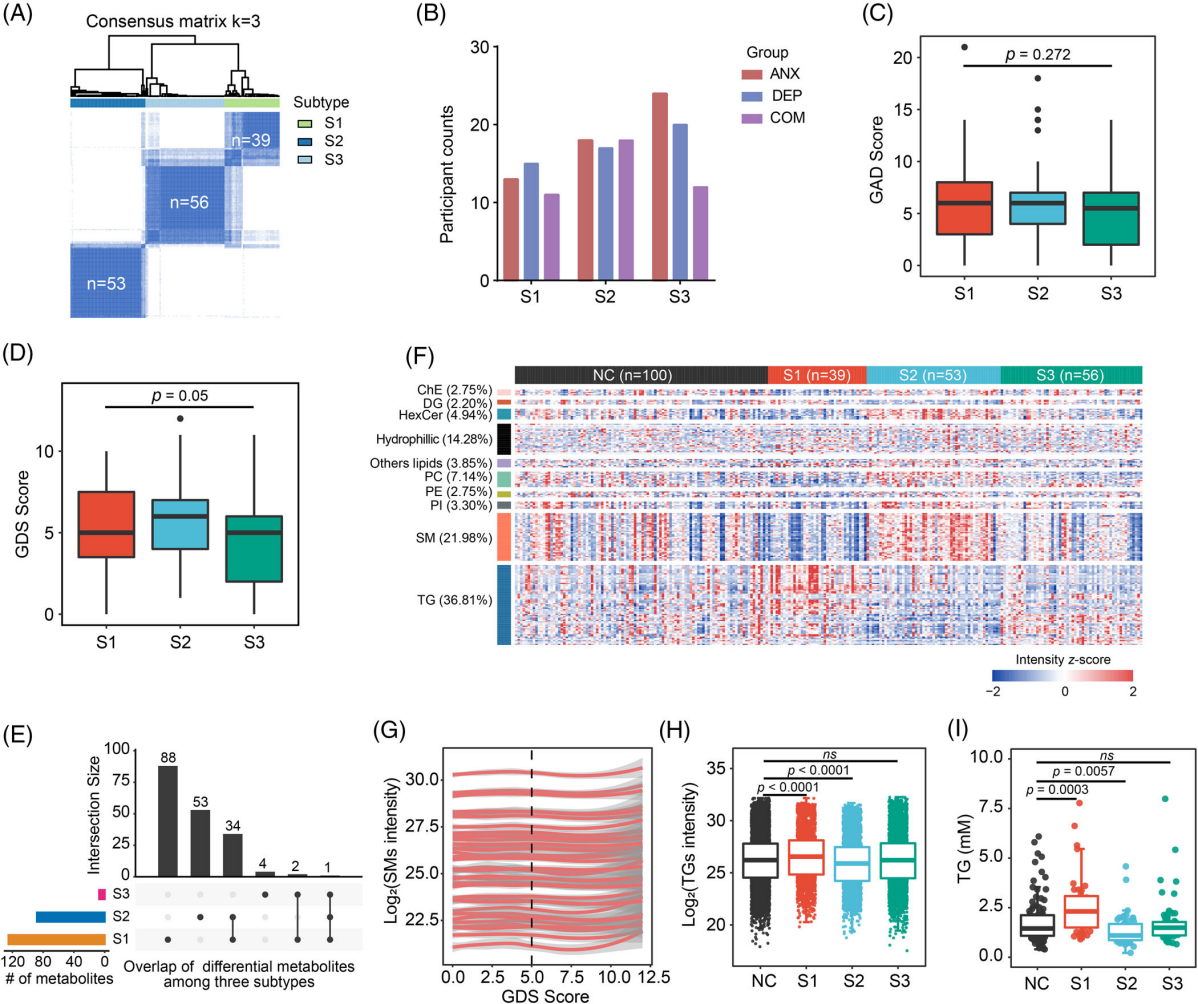
Molecular classification of individuals with mental illnesses. (A) Consensus matrix of *k* = 3 clustered by lipidomics data for 148 individuals with mental disorders. (B) The disease composition of metabolic subtypes. The *y*‐axis represents the counts of participants diagnosed with ANX, DEP, and COM. (C,D) The statistics of GAD score (C) and GDS score between S1 and S3 or S2 and S3. The significance between two subtypes was tested by unpaired Student's *t*‐test. (E) The overlap of significantly changed (Mann–Whitney *U*‐test, *p* < 0.05 and FC (subtype/NC) > 1.25 or < 0.8) metabolites between each subtype and normal controls. (F) Heatmap of the significantly changed metabolites showed in (E). The types of these metabolites are denoted in the left. Heatmap color represents the *z* score intensity of metabolites; red indicates high relative intensity, and blue indicates low relative intensity. (G) SM trajectories with the increase of the GDS score. Plasma SM levels were log2 transformed, and the trajectories of the 40 SMs were estimated by LOESS regression. (H) The distribution of significantly changed TGs, showed in (E), among normal and subtypes. Test: Mann–Whitney *U*‐test. (I) The levels of TG, measured by blood biochemical test, in normal and three metabolic subtypes. The significance is tested by unpaired Student's *t*‐test. ChE: cholesterol ester; DG: diacylglycerol; HexCer: ceramide coupling galactose series; PC: phosphatidylcholine, PE: phosphatidylethanolamine; PI: phosphatidylinositol; SM: sphingomyelin; TG: triacylglycerol; NC, normal control; ANX, anxiety; DEP, depression; COM, comorbid anxiety–depressive

People diagnosed with different mental disorders may have similar molecular profiles and symptoms.^39^ Molecular classification, clustering the individuals using the omics data, allows the identification of many disease‐related signatures that are difficult to be obtained by logistic regression analysis, because of the heterogeneity of the disease.^40^ Moreover, the patients in the same cluster may benefit from the same treatments. To find the metabolic subtypes for individuals with mental disorders, we performed unsupervised classification with lipidomic data, and separated the 148 participants in subcohort1 with anxiety, depression, or comorbid anxiety‐depressive disorder into three clusters (S1, S2, and S3) (Figure 4A and Figure S1A–H).^41^ Statistics showed that subtype S2 had more participants with comorbid anxiety‐depressive disorder, while S3 contained more anxiety persons (Figure 4B). There was no significant difference in the GAD scores among the three subtypes (Figure 4C), but the GDS scores of individuals in S2 were significantly higher than those of participants in S3 (Student's *t*‐test, *p* = 0.0148, Figure 4D).

Then, we used Mann–Whitney *U*‐test to screen the differential metabolites for each subtype compared with normal controls. As a result, a total of 182 differential metabolites were identified, including 125 differential metabolites for S1, 88 differential metabolites for S2, and 7 differential metabolites for S3 (Figure 4E, Mann–Whitney *U*‐test*, p* < 0.05 and FC (subtype/NC) > 1.25 or < 0.8). Among them, 34 metabolites were differentially regulated in both S1 and S2 subtypes. Further analysis showed that 85.7% of the 182 metabolites with significant differences were lipids, of which triglycerides (TGs) and sphingomyelin (SM) dominated. SM, PC, and hexosylceramides (HexCer) decreased in S1 but dramatically increased in S2 (Figure 4F). Besides, the dynamic changes in SM were very interesting. The levels of SM gradually deceased as the GDS score increased from 5.0 to 7.5, but started to increase when the GDS score reached 7.5 and more (Figure 4G). Unlike SMs, TGs were significantly increased in S1 but dramatically decreased in S2 and with no significant changes in S3 compared with normal controls (Figure 4H), which was further validated by blood biochemistry examination (Figure 4I). In conclusion, molecular stratification reveals more metabolic characteristics of mental disorders in older adults.

### 2.4 Clinical relevance of metabolic subtypes

To gain more insights into metabolic subtypes, we next compared their differences in clinical indicators. Several quality of life indicators, including the physical health component score (PCS), mental health component score (MCS), social support rating scale (SSRS), Pittsburgh sleep quality index (PSQI) score and cognitive disorder rate, as well as blood test parameters, including red blood cell count (RBC), hemoglobin (HGB), albumin–globulin (A/G), TG, high‐density lipoprotein (HDL), low‐density lipoprotein (LDL), and very low‐density lipoprotein (VLDL), showed significant differences among disease subtypes and the normal group (*p* < 0.05, Table 2). For example, participants in S3, whose metabolic profile and blood biochemical parameters were similar to those of the normal group (Figure 4F and Table 2), had the highest quality of life. Participants in S1 had lower PCS (53.3 ± 19.0) and higher levels of TG (2.57 ± 1.55). Moreover, more participants in S1 suffered from cognitive disorder (42.9%). Participants in S2 had the lowest MCS (62.5 ± 18.4), SRSS (37.5 ± 8.31), and worst sleep quality (PSQI = 8.53 ± 4.53). HGB (150 ± 16.9) and RBC (4.99 ± 0.47), potential biomarkers of major depressive disorder, increased in S2.^42^ In addition, participants in S1 and S2 are older than those in S3. These results collectively indicate that participants with similar metabolic profiles may have similar mental health conditions.

**TABLE 2:** Clinical relevance of metabolic subtypes

**Subtype**	**Control (*N* = 100)**	**S1 (*N* = 39)**	**S2 (*N* = 53)**	**S3 (*N* = 56)**	***p*‐Value**
Age^a^, mean (SD)	68.8 (10.0)	69.1 (9.23)	69.3 (9.43)	66.9 (8.99)	0.533
BMI^a^, mean (SD)	23.1 (3.12)	23.8 (3.74)	22.7 (3.69)	23.0 (3.06)	0.527
The quality of life					
PCS^a^, mean (SD)	79.2 (13.1)	53.3 (19.0)	56.0 (19.0)	64.7 (18.8)	< 0.001
MCS^a^, mean (SD)	83.3 (10.9)	64.2 (18.0)	62.5 (18.4)	71.2 (13.4)	< 0.001
SRSS^a^, mean (SD)	43.3 (6.80)	38.9 (7.14)	37.5 (8.31)	40.6 (9.16)	< 0.001
PSQI^a^, mean (SD)	5.81 (3.11)	7.54 (3.52)	8.53 (4.53)	7.93 (4.10)	< 0.001
Cognitive disorder^b^,					
No. (%)					<0.001
No	95 (95.0%)	24 (57.1%)	39 (78.0%)	44 (78.6%)	
Yes	5 (5.00%)	18 (42.9%)	11 (22.0%)	12 (21.4%)	
Blood parameters					
RBC^a^, mean (SD)	4.77 (0.55)	4.70 (0.56)	4.99 (0.47)	4.59 (0.59)	0.002
HGB^a^, mean (SD)	144 (14.7)	142 (18.6)	150 (16.9)	138 (17.5)	0.004
A/G^a^, mean (SD)	1.60 (0.23)	1.55 (0.25)	1.52 (0.20)	1.64 (0.24)	0.048
TG^a^, mean (SD)	1.78 (1.16)	2.57 (1.55)	1.32 (0.76)	1.75 (1.21)	< 0.001
HDL^a^, mean (SD)	1.35 (0.36)	1.13 (0.24)	1.52 (0.34)	1.26 (0.25)	< 0.001
LDL^a^, mean (SD)	2.62 (0.79)	2.44 (0.63)	2.85 (0.91)	2.43 (0.68)	0.019
VLDL^a^, mean (SD)	0.81 (0.53)	1.17 (0.71)	0.60 (0.35)	0.80 (0.55)	< 0.001

^a^ Continuous variables, ANOVA test.

^b^ Categorical variables, Chi‐squared test. BMI: body mass index, calculated as weight in kilograms divided by height in meters squared; PCS: physical health component score; MCS: mental health component score; SRSS: social support rating scale; PSQI: Pittsburgh sleep quality index; RBC: red blood cell; HGB: hemoglobin; A/G: albumin‐globulin; TG: triglyceride; HDL: high‐density lipoprotein; LDL: low‐density lipoprotein; VLDL: very low‐density lipoprotein.

### 2.5 Age‐related risk metabolites for depressive symptoms

**FIGURE 5:**
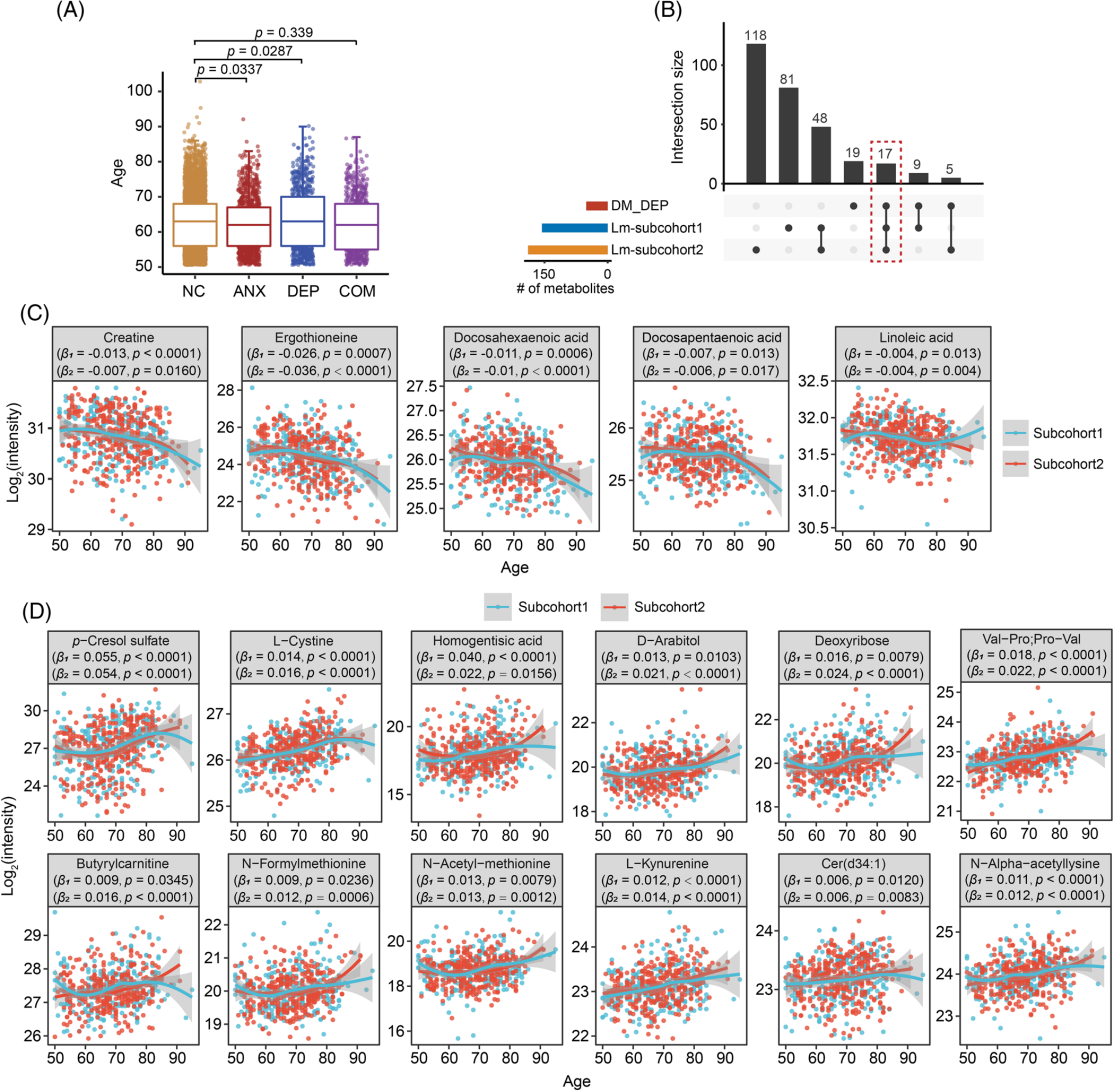
Age‐related metabolites for depressive symptoms. (A) Age distribution of normal, anxiety, depression, and comorbidity in individuals aged over 50 from West China Health and Aging Trend study (WCHAT). The significance was calculated by unpaired Student's *t*‐test. (B) Overlap of the differential metabolites for depression and age‐related metabolites (multiple linear regression, *p* < 0.05) in the two subcohorts. The linear regression model for subcohort1 was adjusted by mental disorder, sex and body mass index (BMI), and for subcohort2 was adjusted by sex and BMI. (B) Seventeen depression‐associated metabolites were correlated with age in the two subcohorts. (C,D) Metabolite trajectories with age. Five metabolites with lower ORs were negatively associated with age (C), and 12 metabolites with higher ORs were positively correlated with age (D). DM_DEP, differential metabolites between normal and depression; Lm, multiple linear regression; NC, normal control; ANX, anxiety; DEP, depression; COM, comorbid anxiety–depressive.

Interestingly, based on the statistics of 6,467 participants over 50 years old from the WCHAT cohort, we found that individuals suffering from depression were significantly older than normal people (Student's *t*‐test, *p* = 0.028, Figure 5A). To identify whether the depression‐associated metabolites were age‐correlated, we applied multiple linear regression to identify metabolites whose levels were correlated with age in subcohort1 and identified 155 significantly correlated metabolites (*p* < 0.05). To confirm these results, we used another independent subcohort2 containing 328 mental health individuals from WCHAT cohort. Multiple linear regression analysis identified 188 age‐associated metabolites (*p* < 0.05). Overlapping analysis revealed that 17 age‐associated metabolites were identified as potential risk factors for depressive symptoms (Figure 5B and Table S1), of which five metabolites had a lower OR for depressive symptoms and were positively correlated with age, including creatine (*β*_1_ = −0.013, *p* < 0.0001; *β*_2_ = −0.007, *p* = 0.0160), ergothioneine (*β*_1_ = −0.026, *p* = 0.0007; *β*_2_ = −0.036, *p* < 0.0001), DHA (*β*_1_ = −0.011, *p* = 0.0006; *β*_2_ = −0.01, *p* < 0.0001), DPA (*β*_1_ = −0.007, *p* = 0.013; *β*_2_ = −0.006, *p* = 0.017), and linoleic acid (*β*_1_ = 0.055, *p* < 0.0001; *β*_2_ = 0.055, *p* < 0.0001, Figure 5C). Of the 17 age‐associated risk metabolites for depressive symptoms, 12 had a higher OR for depressive symptoms, and their levels were positively correlated with age, such as *p*‐cresol sulfate (log_2_(OR) = 0.28, 95% CI: 0.01–0.58, *p* = 0.0484; *β*_1_ = 0.055, *p* < 0.0001; *β*_2_ = 0.055, *p* < 0.0001), L‐cystine (*β*_1_ = 0.014, *p* < 0.0001; *β*_2_ = 0.016, *p* < 0.0001), homogentisic acid (*β*_1_ = 0.040, *p* < 0.0001; *β*_2_ = 0.022, *p* = 0.0156), D‐Arabitol (*β*_1_ = 0.013, *p* = 0.0103; *β*_2_ = 0.021, *p* < 0.0001), and L‐kynurenine (*β*_1_ = 0.012, *p* < 0.0001; *β*_2_ = 0.014, *p* < 0.0001, Figure 5D).

**FIGURE 6:**
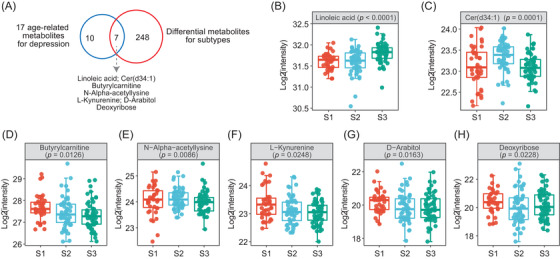
Age‐related metabolites for metabolic subtypes. (A) Overlap of 17 age‐ and depression‐related metabolites, and 255 differential metabolites among three subtypes (Kruskal–Wallis test, *p* < 0.05). (B–H) The distribution of linoleic acid (B), Cer(d34:1) (C), butyrylcarnitine (D), N‐alpha‐acetyllysine (E), L‐kynurenine (F), D‐arabitol (G), and deoxyribose (H) among three metabolic subtypes. Test: Kruskal–Wallis test

To explore whether the above 17 age‐ and depression‐associated metabolites were differentially regulated in three metabolic subtypes, overlapping analysis between the 17 metabolites and the 255 metabolites that significantly varied among the three subtypes (Kruskal–Wallis test, *p* < 0.05) was performed. As a result, seven metabolites including linoleic acid, Cer(d34:1), butyrylcarnitine, *N*‐alpha‐acetyllysine, L‐kynurenine, D‐arabitol, and deoxyribose were obtained (Figure 6A and Table S2). Linoleic acid is reduced in depression and higher in S3, but the other six metabolites showed the opposite trend (Figure 6B–H). For example, Cer(d34:1), which has a higher risk in people with depression (log_2_(OR) = 1.66, 95% CI: 0.19–3.23, *p* = 0.0308) and is positively associated with age (*β*_1_ = 0.006, *p* = 0.0120; *β*_2_ = 0.006, *p* = 0.0083), was significantly increased in S2, which indicates that the increase in Cer(d34:1) may be an important signature for geriatric depression (Figure 6C). Together, we identified some age‐related metabolites as potential risk factors for depressive symptoms.

## 3 DISCUSSION

In this study, based on a prospective multi‐center cohort WCHAT, we investigated the association between dysregulated metabolism and mental disorders and the connection between age and depression. Several interesting findings were gained. First, we presented the potential metabolic risk factors for anxiety, depression, and comorbid anxiety‐depressive disorder for older adults in Western China. The more severe the mental disorders were, the more dysregulated metabolites, especially lipids, were in their blood. Second, we disclosed that individuals with different mental disorders but close metabolic profiles most likely had similar clinical phenotypes, and the metabolic profile might serve as a progression indicator of mental illnesses. Third, we confirmed that patients who suffered from geriatric depression were older than normal people, and 17 age‐associated metabolites were identified as potential risk factors for depressive symptoms. Among them, five metabolites negatively correlated with age showed a lower risk for geriatric depression, including creatine, ergothioneine, DHA, DPA, and linoleic acid. Twelve metabolites, such as *p*‐cresol sulfate, homogentisic acid, D‐arabitol, Cer(d34:1), and kynurenine, were positively associated with age and showed a higher risk for geriatric depression.

Inflammaging is associated with many age‐related disorders, including geriatric depression, probably induced by pronounced and prolonged immune responses.^43^, ^44^ The imbalance of many endogenous proinflammatory and anti‐inflammatory chemicals is associated with mental disorders. For example, benzenebutanoic acid ameliorates lipopolysaccharide‐induced anxiety and depressive‐like behavior by reducing oxidation stress and the neuroinflammatory cascade.^45^, ^46^ 1‐Methylnicotinamide, a main metabolite of nicotinamide, is anti‐inflammatory and can ameliorate chronic unpredictable mild stress (CUMS)‐induced depression.^47^, ^48^ In addition, polyunsaturated fatty acids (PUFAs) are important in regulating redox balance. Dietary supplementation with omega‐3 PUFAs, such as linolenic acid, EPA, DHA, and DPA, can prevent geriatric depression,^49^ probably by binding to mediators of inflammation, such as PPARγ, resolvins, and GPR120.^50^ A previous study demonstrated that a high level of serum linoleic acid, an omega‐6 PUFA, may be protectively associated with depression.^51^ In the present study, a reduction in plasma linoleic acid was not only a potential risk factor for geriatric depressive symptoms but also for anxiety and comorbid anxiety‐depressive symptoms. The mechanism by which linoleic acid protects mental health may require more investigation. In addition, the antidepressants and horticultural therapy are also useful methods to reduce proinflammatory cytokines and improve mental health.^28^, ^52^

Unlike hydrophilic metabolites,^21^, ^53^ the relevance of lipid imbalance to mental illnesses is still less understood. Metabolic classification in this study discloses obvious lipid dysregulation occurring in severe mental disorders. Comparative analysis revealed that S1 and S2 are metabolism‐associated subtypes with severe mental illnesses, while S3 is metabolism‐independent with mild mental disorders. SM is a major sphingolipid that is specifically enriched in membranes of mammalian cells.^54^ Dysregulated transformation of SM to ceramide (Cer) may induce mental disorders.^55^ The antidepressants amitriptyline and fluoxetine can reduce the activity of ASM and the concentration of Cer in the hippocampus.^56^ In this study, we found that the abundance of SMs were distinct in metabolic subtypes and changed in a wave‐like manner in participants as GDS score increased. In the early stage of depression, SM decreases, and the production of myelin becomes less sufficient, which may impair cognition. As the progression of depression worsens, SM starts to increase, leading to increased downstream metabolites such as HexCer and Cer(d34:1), which may further accelerate mental illness. Moreover, increased SM may also induce the occurrence of other comorbid diseases, such as CAD, reducing people's quality of life.^57^ Therefore, due to distinct profiles of SM and Cer in patients, precise treatment by inhibiting the activity of ASM to reduce Cer might be more effective in patients with high Cer.

TG is another kind of lipid showing distinct levels among metabolic subtypes. The levels of blood TGs in S1 participants were very high, while the situation was reversed in S2. People in the S1 subgroup had higher GDS scores, worse physical health, and suffered more from cognitive impairment, which may be associated with the abnormal increase in TGs. Elevated levels of TGs in blood are associated with unstable blood sugar levels and insulin resistance, both of which are risk factors for diabetes.^58^ Diabetes and mental health are tightly connected and interact,^59^, ^60^ and both type 1 and type 2 diabetes can increase the risk of depression. Moreover, elevated serum concentrations of TGs are also risk factors for cognitive impairment associated with leptin resistance.^61^, ^62^

People with mental disorders usually have psychosocial functioning limitations, poor physical function, and diminished quality of life.^63^ However, questionnaires such as the GDS‐15 or GAD‐7 underestimate these issues.^64^ Based on the clinical statistics of metabolic subtypes, individuals in S1 and S2 suffer greatly from these issues, while individuals in S3 have a much better quality of life, which indicates that metabolic changes could reflect the severity of mental disorders. If a person is diagnosed with mild mental illness but his/her metabolic profile is similar to that of the population with more severe mental disorders, doctors should pay more attention to his/her overall health. In a word, similar metabolic profiles are associated with close clinical phenotypes such as quality of life, social support, sleep quality, and cognitive levels in participants with mental disorders. The severer the mental disorder is, the more dysregulated metabolites could be found. The mental health assessment may be more accurate if the metabolic profile of an individual is considered.

Some metabolites that increase with age, such as *p*‐cresol sulfate, homogentisic acid, and kynurenine, are proinflammatory chemicals associated with a higher risk of geriatric depression.^65^, ^66^, ^67^, ^68^ The increase in homogentisic acid, a metabolic disorder that ultimately prompts the development of inflammatory arthritis by generating O_2_^−^, H_2_O_2_, and •OH by autoxidation,^69^ may contribute to high oxidation stress and exacerbate depressive symptoms in older adults. Homogentisic acid oxidase is an enzyme involved in the catabolism of homogentisic acid. Increasing the enzyme activity of homogentisic acid oxidase may be beneficial for alleviating depressive symptoms.^70^ Besides, there is growing evidence indicating that the kynurenine pathway is involved in the pathophysiology of depression.^71^ Kynurenine is a metabolite of tryptophan catalyzed by tryptophan 2,3‐dioxygenase (TDO) and indoleamine 2,3‐dioxygenase (IDO1).^72^ Inflammation‐induced activation of IDO1 by proinflammatory cytokines may lead to an age‐related increase in kynurenine.^72^ An imbalance in the kynurenine metabolic pathway is associated with the incidence of depression. In major depressive disorder, peripheral kynurenine is preferentially converted to neurotoxin and quinolinic acid rather than neuroprotective kynurenic acid.^73^ *p*‐Cresol sulfate is a protein‐bound uremic toxin derived from tyrosine and phenylalanine in the liver and gut. Because of the albumin‐binding capacity, the gut microbiota metabolite *p*‐cresol sulfate accumulates in the blood during renal function decrease and aging.^74^, ^75^ Circulating *p*‐cresol sulfate increases oxidative stress and neuroinflammation, which may be a cause of depression.^76^

There are still some limitations that should be noted in this study. First, the metabolic disturbance that identified in mental disorders should be validated in another geriatric population. Second, metabolic disturbance identified in this study for mental illness should be further confirmed in brain tissues, which could promote the investigation of their functions for the development of disease. Third, more examination should be concentrated on the application of metabolic classification for the assessment of mental illness. Besides, many other metabolites also increased with age and were associated with a higher risk of depressive symptoms, such as D‐arabitol, deoxyribose, and N‐alpha‐acetyllysine, but their roles and mechanisms in the development of depression are unclear.

## 4 MATERIALS AND METHODS

### 4.1 Participants and study design

WCHAT is an ongoing population‐based cohort study of community‐dwelling older adults aged over 50 in Western China. The depressive and anxiety symptoms of participants were assessed by two doctors through the GDS‐15 and GAD‐7 questionnaire, respectively, with a threshold score of 5.^77^, ^78^, ^79^ The demographic and clinical variables assessed by two doctors using the structured questionnaire, included age, sex, smoking, drinking wine, PCS, MCS, SSRS, and PSQI. Height and weight were measured on a height scale (CSTF‐5000) and bioelectrical impedance analysis (Inbody 770), respectively. BMI was calculated as weight in kilograms divided by height in meters squared.

A total of 6,467 participants met the inclusion criteria: (1) age > 50 years; (2) no missing data on depression or anxiety; and (3) having human plasma samples at baseline in the WCHAT study. Among them, 248 WCHAT participants, including 41 individuals with comorbid anxiety‐depressive disorder (GAD score > 5 and GDS score > 5), 52 persons with depression (only GDS score > 5), 55 cases with anxiety (only GAD score > 5), and 100 control participants (GDS score and GAD score are less than 1), without tumor and other severe disease, were randomly selected for this study. To validate age‐related metabolites, we sampled an additional 328 mentally healthy individuals, which were also without tumor and other severe disease, from WCHAT (GDS score < 5 and GAD score < 5).

Fasting blood samples were collected in EDTA‐coated 10 ml tubes. Plasma was isolated by centrifugation at 1600 ×*g* for 10 min at 4°C. The supernatant was divided into equal volumes, transferred into liquid nitrogen for 3 min, and finally stored at −80°C until LC–MS/MS analysis.

### 4.2 Extraction of metabolites and lipids

Hydrophilic metabolites were extracted from plasma using four volumes of methanol, and lipids were extracted with liquid–liquid extraction by adding dichloromethane/methanol (v/v = 2:1). Untargeted metabolomics and lipidomics were carried out at the Facility Center of Metabolomics and Lipidomics of Tsinghua University.^80^, ^81^, ^82^

The plasma samples were centrifuged at 14,000 ×*g* for 20 min at 4**°**C, and then 90 μl of plasma was used for the extraction of hydrophilic metabolites and lipids. QC samples consisted of mixed plasma samples. ^13^C_6_ L‐Lysine hydrochloride powder (Silantes) and ^13^C_6_^15^N_4_ L‐arginine hydrochloride powder (Silantes) were added as the internal standards for the hydrophilic metabolites. PE (16:0‐d31‐18:1) was added as the internal standard for the lipids.

Polar metabolites were extracted using prechilled methanol (MS grade) according to the method of Yuan et al.^83^ After adding 360 μl of methanol, the samples were vortexed and stored at −80**°**C for 1 h and then centrifuged at 14,000 ×*g* for 10 min. Equal volumes of supernatant were collected and transferred to a new tube and finally dried in a vacuum centrifuge. To monitor the performance of data acquisition, Gln‐N_15_, inosine‐^4^N_15_, and Trp‐D_5_ were added as internal standards for the positive mode, and cholic acid‐D_4_, inosine‐^4^N_15_, stearic acid‐D_35_, succinate‐D_4_, and Trp‐D_5_ were added as internal standards for the negative mode before LC–MS/MS analysis.

Lipids were extracted according to the method of Bligh and Dyer.^84^ The plasma was extracted by adding 360 μl of prechilled dichloromethane/methanol mixture (v:v = 2:1, MS grade). After enough vortex, the samples were centrifuged at 300 rpm for 15 min at 4**°**C. Subsequently, the lower dichloromethane layer was collected and transferred to a new tube. Then, prechilled water (MS grade) (1/5 volume of dichloromethane) was added for re‐extraction and centrifuged at 10,000 rpm for 20 min at 4**°**C. The lower dichloromethane layer containing lipids was collected and dried in speedvacuum. D_31_‐Cer(d18:1_16:0), DG(14:0_14:0), PC(14:0_14:0), PE(14:0_14:0), D_31_‐PE(16:0_18:1), and PS(14:0_14:0) were added as internal standards before LC–MS/MS analysis.

### 4.3 Mass spectrometry analyses of polar metabolites and lipids

Mass spectrometry analyses of polar metabolites and lipids were carried out by the Facility Center of Metabolomics and Lipidomics of Tsinghua University, according to the method described by Tang et al.^85^ Metabolomics analysis was performed using a BEH Amide column (Waters, USA) in the positive ion mode and a BEH C18 column (Waters, USA) in the negative ion mode. Lipidomics was performed using a CORTECS C18 column (Waters, USA). Pooled QCs for metabolomics and lipidomics were inserted for every 15–20 injections of plasma samples. Polar metabolites were assigned using TraceFinder (Thermo, CA) based on an in‐house database. Standard MS/MS spectra of over 1,500 metabolites were included in the database. Lipids were identified using Lipidsearch (Thermo, CA) software. Only lipids with reliable MS/MS were considered in the subsequent statistical analysis.

### 4.4 Statistical analysis

After filtering the metabolites undetected in > 20% of samples and with a coefficient of variation (CV) of QCs > 30%, we normalized the total intensities of all samples. The missing values were imputed by *K*‐nearest neighbor algorithms (R package: NormalizeMets). T‐distributed stochastic neighbor embedding (t‐SNE) analysis was applied to evaluate the quality of the data. To determine the differential metabolites between normal and anxiety, depression, or comorbid anxiety‐depressive disorder, we built a multiple logistic regression model shown below:

$$\begin{array}{ccc} \mathrm{Mental}\;\mathrm{disorder} & ∼ & \alpha +\beta 1\;\mathrm{metabolite}\;\mathrm{level}+\beta 2\;\mathrm{age}\\ & & +\;\beta 3\;\mathrm{sex}+\beta 4\;\mathrm{BMI}. \end{array}$$

We defined metabolites with *p* < 0.05 between psychiatric patients and normal individuals as significantly changed metabolites.

Then, we used multiple linear regression to explore the age‐related metabolites for the two subcohorts. The models are shown below. Subcohort1, Metabolite level ∼ α+ *β*1 mental disorder + *β*2 age + *β*3 sex + *β*4 BMI; subcohort2, metabolite level ∼ α+ *β*1 age + *β*2 sex + *β*3 BMI. We defined metabolites with *p* < 0.05 as those significantly associated with age.

Consensus clustering of lipidomics data was carried out to determine the metabolic subtypes of participants with anxiety and/or depression and to seed random (R package: ConsensusCluster Plus).^86^ The top 150 lipids that varied among the 148 individuals were used for *k*‐means clustering. Up to eight clusters were tested, and the distance was measured by Euclidean distance. The consensus matrices for *k* = 2–8 are shown in Figure S1A–G. Clustering with *k* = 3 had the lowest proportion of ambiguous clustering (PAC), and subtypes were separated clearly, which means *k* = 3 is the optimal cluster. In addition, the cumulative distribution function (CDF) curve showed that when *k* = 3, the descent gradient was the minimum (Figure S1H). Taken together, metabolic subtypes of mental disorders were defined by *k*‐means consensus clustering with an optimal *k* = 3.

Normality tests revealed that the metabolites were almost skewed in the distribution. Therefore, the significance of metabolites between subtypes and normal were tested by the Mann–Whitney *U*‐test. Significantly different metabolites in metabolic subtypes were screened with *p* < 0.05. FC (subtype/NC) > 1.25 or < 0.8. In addition, we applied the Kruskal–Wallis test to identify the significantly changed metabolites among the three subtypes (*p* < 0.05). Clinical information and blood biochemical indexes for participants were normally distributed and tested by parametric tests. Unpaired Student's *t*‐test and one‐way ANOVA test were used for the continuous variables of two groups and multiple groups, respectively. Classified variables were tested by the Chi‐squared test.

## CONFLICT OF INTEREST

The authors declare that there is no conflict of interest that could be perceived as prejudicing the impartiality of the research reported.

## ETHICS APPROVAL

The subjects in this research are from WCHAT cohort. The Ethics Committee of Sichuan University approved this study (Permission Number: 2017‐445), and all procedures were conducted based on the principles of the Declaration of Helsinki. All the participants have signed the informed consents to conduct this study.

## AUTHOR CONTRIBUTIONS

Birong Dong, Lunzhi Dai, and Biao Dong designed the project; Yu Liu, Wanyu Zhao, Ying Lu, Yunli Zhao, Yan Zhang, and Miao Dai participated in the sample collection; Yu Liu, Wanyu Zhao, Yunli Zhao, and Ying Lu processed the sample preparation and data analysis; Lunzhi Dai, Biao Dong, Yu Liu, and Wanyu Zhao drafted and revised the manuscript; Peng Lei, Jirong Yue, Shan Hai, Ning Ge, Shuting Zhang, Mingjin Huang, Xiaohui Liu, and Shuangqing Li provided technical supports and suggestions.

## Supporting information



Supporting Information

## Data Availability

The data used in this study were included in the Supplementary Information.
