# Correction to “Systematic Metabolic Characterization of Mental Disorders Reveals Age‐related Metabolic Disturbances as Potential Risk Factors for Depression in Older Adults”

**DOI:** 10.1002/mco2.71076

**Published:** 2026-10-22

Liu Y, Zhao W, Lu Y, et al. Systematic metabolic characterization of mental disorders reveals age‐related metabolic disturbances as potential risk factors for depression in older adults. *MedComm*. 2022; 3:e165, https://doi.org/10.1002/mco2.165.

In the process of checking the original data, the authors identified inadvertent errors in TABLE 1 and TABLE 2, as well as in the results descriptions in the original manuscript. The marital status data and BMI value for Subcohort 2 in TABLE 1 and the numbers and percentages of cognitive disorder cases in S1 and S2 in TABLE 2 were incorrectly reported. The correct tables and descriptions are shown below. The authors apologize for these oversights and declare these corrections do not affect the conclusions of the study.

**TABLE 1:** Clinical characteristics of participants in this study.

	Subcohort1					Subcohort2
	Control	Anxiety	Depression	Comorbid	*p*‐value	Normal
	(N=100)	(N=55)	(N=52)	(N=41)		(N=328)
GDS score^a^, mean (SD)	0.5 (0.5)	2.5 (1.2)	6.3 (1.6)	7.7 (2.2)	< 0.001	2.0 (1.2)
GAD score^a^, mean (SD)	0.1 (0.3)	7.5 (2.6)	1.9 (1.6)	8.9 (4.2)	< 0.001	1.1 (1.3)
Age^a^, mean (SD)	68.8 (10.0)	69.6 (8.3)	68.7 (9.9)	66.1 (9.3)	0.33	68.5 (8.7)
Sex^b^, No. (%)					0.27	
Female	62 (62.0%)	38 (69.1%)	39 (75.0%)	31 (75.6%)		201 (61.3%)
Male	38 (38.0%)	17 (30.9%)	13 (25.0%)	10 (24.4%)		127 (38.7%)
Education^b^, No. (%)					0.39	
Illiterate	27 (27.0%)	21 (38.2%)	21 (40.4%)	13 (31.7%)		104 (31.7%)
Primary school	40 (40.0%)	19 (34.5%)	21 (40.4%)	13 (31.7%)		129 (39.3%)
Secondary school and above	33 (33.0%)	15 (27.3%)	10 (19.2%)	15 (36.6%)		95 (29.0%)
Marital status^b^, No. (%)					0.97	
Married	71 (71.0%)	41 (74.5%)	37 (71.2%)	30 (73.2%)		259 (79.0%)
Unmarried/Widowed/Divorced	29 (29.0%)	14 (25.5%)	15 (28.8%)	11 (26.8%)		69 (21.0%)
Smoking^b^, No. (%)					0.44	
No	79 (79.0%)	46 (83.6%)	44 (88.0%)	36 (87.8%)		251 (76.8%)
Yes	21 (21.0%)	9 (16.4%)	6 (12.0%)	5 (12.2%)		76 (23.2%)
Drinking wine^b^, No. (%)					0.33	
No	85 (85.0%)	44 (80.0%)	48 (92.3%)	34 (82.9%)		251 (76.5%)
Yes	15 (15.0%)	11 (20.0%)	4 (7.69%)	7 (17.1%)		77 (23.5%)
BMI^a^, mean (SD)	23.1 (3.1)	24.1 (3.6)	23.1 (3.5)	21.8 (3.0)	0.009	23.2 (3.12)

**TABLE 2:** Clinical relevance of metabolic subtypes

Subtype	Control (N=100)	S1 (N=39)	S2 (N=53)	S3 (N=56)	*p*‐value
Age^a^, mean (SD)	68.8 (10.0)	69.1 (9.23)	69.3 (9.43)	66.9 (8.99)	0.533
BMI^a^, mean (SD)	23.1 (3.12)	23.8 (3.74)	22.7 (3.69)	23.0 (3.06)	0.527
The quality of life					
PCS^a^, mean (SD)	79.2 (13.1)	53.3 (19.0)	56.0 (19.0)	64.7 (18.8)	< 0.001
MCS^a^, mean (SD)	83.3 (10.9)	64.2 (18.0)	62.5 (18.4)	71.2 (13.4)	< 0.001
SRSS^a^, mean (SD)	43.3 (6.80)	38.9 (7.14)	37.5 (8.31)	40.6 (9.16)	< 0.001
PSQI^a^, mean (SD)	5.81 (3.11)	7.54 (3.52)	8.53 (4.53)	7.93 (4.10)	< 0.001
Cognitive disorder^b^,					<0.001
No. (%)					
No	95 (95.0%)	22 (56.4%)	41 (77.4%)	44 (78.6%)	
Yes	5 (5.00%)	17 (43.6%)	12 (22.6%)	12 (21.4%)	
Blood parameters					
RBC^a^, mean (SD)	4.77 (0.55)	4.70 (0.56)	4.99 (0.47)	4.59 (0.59)	0.002
HGB^a^, mean (SD)	144 (14.7)	142 (18.6)	150 (16.9)	138 (17.5)	0.004
A/G^a^, mean (SD)	1.60 (0.23)	1.55 (0.25)	1.52 (0.20)	1.64 (0.24)	0.048
TG^a^, mean (SD)	1.78 (1.16)	2.57 (1.55)	1.32 (0.76)	1.75 (1.21)	< 0.001
HDL^a^, mean (SD)	1.35 (0.36)	1.13 (0.24)	1.52 (0.34)	1.26 (0.25)	< 0.001
LDL^a^, mean (SD)	2.62 (0.79)	2.44 (0.63)	2.85 (0.91)	2.43 (0.68)	0.019
VLDL^a^, mean (SD)	0.81 (0.53)	1.17 (0.71)	0.60 (0.35)	0.80 (0.55)	< 0.001

^a^Continuous variables, ANOVA test.

^b^Categorical variables, Chi‐squared test. BMI: body mass index, calculated as weight in kilograms divided by height in meters squared; PCS: physical health component score; MCS: mental health component score; SRSS: social support rating scale; PSQI: Pittsburgh sleep quality index; RBC: red blood cell; HGB: hemoglobin; A/G: albumin‐globulin; TG: triglyceride; HDL: high‐density lipoprotein; LDL: low‐density lipoprotein; VLDL: very low‐density lipoprotein.

The corresponding description in Section 2.4 was revised to “Moreover, more participants in S1 suffered from cognitive disorder (43.6%).” In Section 2.5, the corrected statement is: “of which five metabolites had a lower OR for depressive symptoms and were negatively correlated with age.” For linoleic acid, the corrected description is β1 = −0.004 (*p* = 0.013) and β2 = −0.004 (*p* = 0.004). For *p*‐cresol sulfate, the corrected description of β2 is 0.054. For the WCHAT cohort: “individuals suffering from depression were significantly older than normal people (Student’s *t*‐test, *p* = 0.0287, Figure 5A)”.

