# Supplementary material for: Systematic metabolic characterization of mental disorders reveals age‐related metabolic disturbances as potential risk factors for depression in older adults
Source: MedComm (2020). 2022 Sep 30;3(4):e165. doi: 10.1002/mco2.165 (PMC9523679; doi:10.1002/mco2.165)
Supplement: Supplementary file 1 — Supporting Information [file MCO2-3-e165-s001.docx]

**Systematic metabolic characterization of mental disorders reveals age-related metabolic disturbances as potential risk factors for depression in older adults**

Yu Liu,^1,†^ Wanyu Zhao,^1,†^ Ying Lu,^1,†^ Yunli Zhao,^2,†^ Yan Zhang,^1^ Miao Dai,^1^ Shan Hai,^1^ Ning Ge,^1^ Shuting Zhang,^3^ Mingjin Huang,^4^ Xiaohui Liu,^5^ Shuangqing Li,^1^ Jirong Yue,^1^ Peng Lei,^1^ Biao Dong,^1,^* Lunzhi Dai,^1,^* Birong Dong,^1,^*

^1^National Clinical Research Center for Geriatrics and Department of General Practice, State Key Laboratory of Biotherapy, West China Hospital, Sichuan University, Chengdu, 610041, China.

^2^Department of Health Research Methods, Evidence, and Impact, McMaster University, Hamilton, Ontario, Canada

^3^Department of Neurology, West China Hospital, Sichuan University, Chengdu, 610041, China.

^4^The Third Hospital of Mianyang, Sichuan Mental Health Center, Mianyang, 612000, China.

^5^School of Life Sciences, Tsinghua University, Beijing, 100084, China.

^†^These authors contributed equally to this work.

*Correspondence: lunzhi.dai@scu.edu.cn (Dr. Lunzhi Dai); birongdong123@outlook.com (MD. Birong Dong); biaodong@scu.edu.cn (Dr. Biao Dong).

**Running title**

Metabolism links age and depression

**Figure S1**


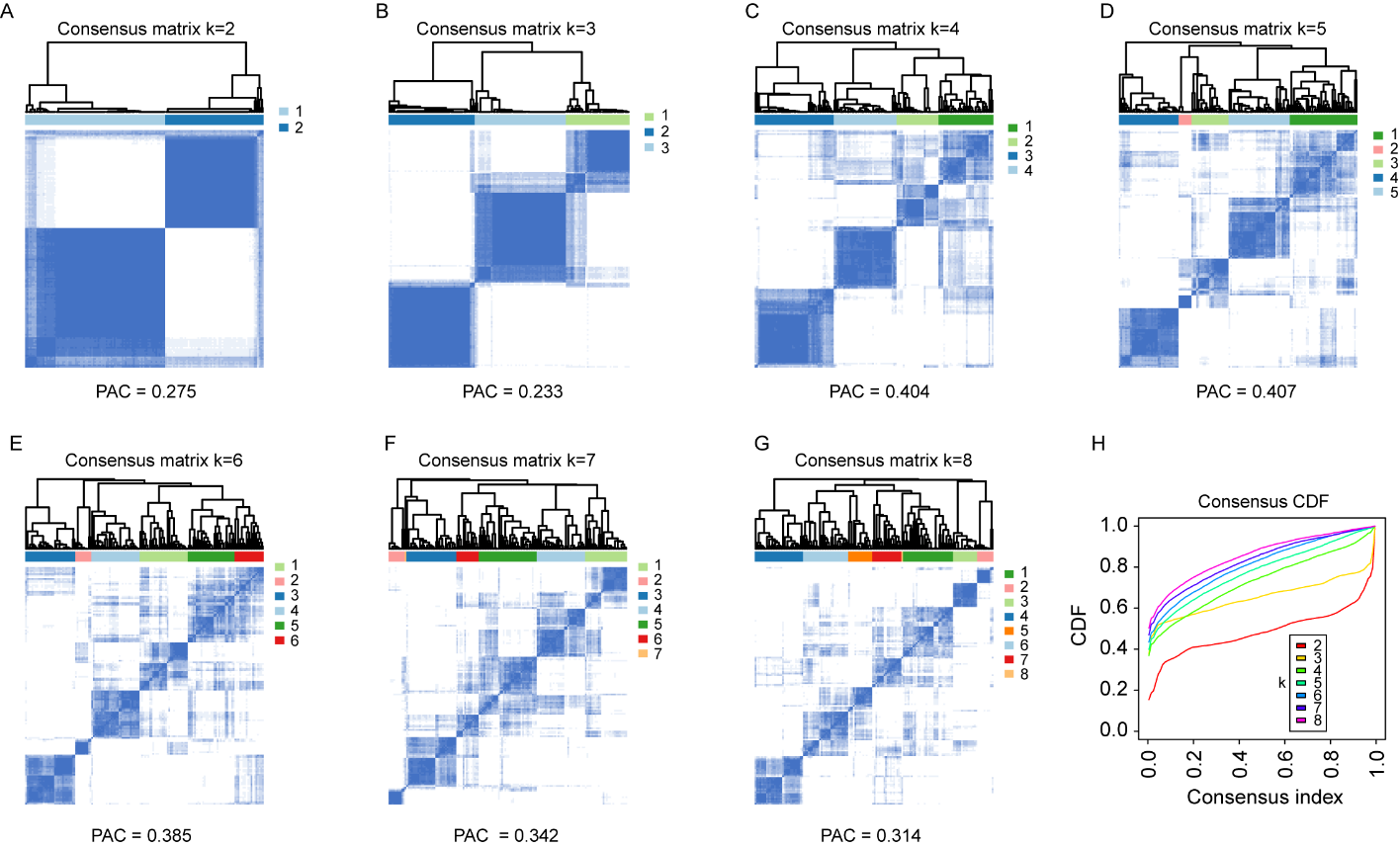


**Figure S1. Unsupervised consensus clustering of 148 individuals into 3 subtypes based on lipidome patterns.** A-G, Consensus matrix of 148 samples from k=2 to k=8. Consensus clustering was performed based on the top 150 lipids that varied among the 148 individuals. The distance was measured by Euclidean distance, and samples were clustered by *k*-means. H, The cumulative distribution function (CDF) plots corresponding to the consensus matrices from k=2 to k=8.

**Table S1. List of 17 age- and depression-associated metabolites.**

|  | Multiple linear regression with age in subcohort1 | | Multiple linear regression with age in subcohort2 | | Multiple logistic regression between normal and depression in subcohort1 | | | |
| --- | --- | --- | --- | --- | --- | --- | --- | --- |
| Metabolites | Coefficient | *p* value | Coefficient | *p* value | OR | 2.5% | 97.5% | *p* value |
| Ergothioneine | -0.026 | 0.0007 | -0.036 | <0.0001 | 0.70 | 0.50 | 0.96 | 0.0289 |
| Creatine | -0.013 | <0.0001 | -0.007 | 0.0165 | 0.30 | 0.11 | 0.74 | 0.0119 |
| DHA | -0.011 | 0.0006 | -0.010 | 0.0001 | 0.32 | 0.14 | 0.70 | 0.0052 |
| DPA | -0.007 | 0.0129 | -0.006 | 0.0174 | 0.36 | 0.14 | 0.87 | 0.0259 |
| Linoleic acid | -0.004 | 0.0128 | -0.004 | 0.0037 | 0.06 | 0.01 | 0.25 | 0.0004 |
| Cer(d34:1) | 0.006 | 0.0120 | 0.006 | 0.0083 | 3.17 | 1.14 | 9.38 | 0.0308 |
| Butyrylcarnitine | 0.009 | 0.0345 | 0.016 | <0.0001 | 1.78 | 1.02 | 3.21 | 0.0469 |
| N-Formylmethionine | 0.009 | 0.0236 | 0.012 | 0.0006 | 1.85 | 1.03 | 3.40 | 0.0407 |
| N-Alpha-acetyllysine | 0.011 | <0.0001 | 0.012 | <0.0001 | 4.04 | 1.45 | 12.54 | 0.0105 |
| L-Kynurenine | 0.012 | <0.0001 | 0.014 | <0.0001 | 2.44 | 1.02 | 6.09 | 0.0487 |
| D-Arabitol | 0.013 | 0.0103 | 0.021 | <0.0001 | 1.61 | 1.03 | 2.59 | 0.0417 |
| N-Acetyl-methionine | 0.013 | 0.0079 | 0.013 | 0.0012 | 1.86 | 1.12 | 3.18 | 0.0189 |
| L-Cystine | 0.014 | <0.0001 | 0.016 | <0.0001 | 5.01 | 1.64 | 16.95 | 0.0065 |
| Deoxyribose | 0.016 | 0.0079 | 0.024 | <0.0001 | 1.61 | 1.09 | 2.44 | 0.0188 |
| Val-Pro;Pro-Val | 0.018 | <0.0001 | 0.022 | <0.0001 | 3.43 | 1.45 | 9.01 | 0.0076 |
| Homogentisic acid | 0.040 | <0.0001 | 0.022 | 0.0156 | 1.39 | 1.07 | 1.83 | 0.0169 |
| *p*-Cresol sulfate | 0.055 | <0.0001 | 0.054 | <0.0001 | 1.22 | 1.01 | 1.49 | 0.0484 |

Note: DHA, Docosahexaenoic acid; DPA: Docosapentaenoic acid.

**Table S2. List of 7 age- and depression-associated metabolites differentially regulated in 3 metabolic subtypes.**

| Metabolites | Ratio (S1/S3) | Ratio (S2/S3) | *p* value | FDR |
| --- | --- | --- | --- | --- |
| Butyrylcarnitine | 1.26 | 1.06 | 0.0126 | 0.0346 |
| L-Kynurenine | 1.21 | 1.00 | 0.0248 | 0.0623 |
| N-Alpha-acetyllysine | 1.07 | 1.07 | 0.0086 | 0.0243 |
| D-Arabitol | 1.50 | 1.03 | 0.0163 | 0.0432 |
| Deoxyribose | 1.26 | 0.92 | 0.0228 | 0.0585 |
| Linoleic acid | 0.88 | 0.87 | <0.0001 | <0.0001 |
| Cer(d34:1) | 1.01 | 1.24 | 0.0001 | 0.0004 |

Note: The significance of metabolites was tested by Kruskal-Wallis test for 3 metabolic subtypes. FDR was corrected by Benjamini & Hochberg. The ratios were calculated using median intensity of each metabolite in the corresponding subtype.
